# Supplementary material for: I love the way you love me: Responding to partner’s love language preferences boosts satisfaction in romantic heterosexual couples
Source: PLoS One. 2022 Jun 22;17(6):e0269429. doi: 10.1371/journal.pone.0269429 (PMC9216579; doi:10.1371/journal.pone.0269429)
Supplement: S1 Appendix — (DOCX) [file pone.0269429.s001.docx]

Table 5. Bivariate correlations between mismatch in each love language (preferred vs. felt), relationship satisfaction, and sexual satisfaction in male participants.

|  | 1. | 2. | 3. | 4. | 5. | 6. |
| --- | --- | --- | --- | --- | --- | --- |
| 1. Acts of Service (mismatch) | --- |  |  |  |  |  |
| 2. Physical Touch (mismatch) | .01 | --- |  |  |  |  |
| 3. Words of Affirmation (mismatch) | .27** | .28** | --- |  |  |  |
| 4. Quality Time (mismatch) | .21* | .44* | .22* | --- |  |  |
| 5. Gifts (mismatch) | .17 | .12 | .17 | .18 | --- |  |
| 6. Relationship Satisfaction (men) | -.11 | -.29** | -.28** | -.46** | -.00 | --- |
| 7. Sexual Satisfaction (men) | -.05 | -.31** | -.27** | -.46** | -.05 | .67** |

Table 6. Bivariate correlations between mismatch in each love language, relationship satisfaction, and sexual satisfaction in female participants.

|  | 1. | 2. | 3. | 4. | 5. | 6. |
| --- | --- | --- | --- | --- | --- | --- |
| 1. Acts of Service (mismatch) | --- |  |  |  |  |  |
| 2. Physical Touch (mismatch) | .13 | --- |  |  |  |  |
| 3. Words of Affirmation (mismatch) | .42** | .42** | --- |  |  |  |
| 4. Quality Time (mismatch) | .26** | .60** | .43** | --- |  |  |
| 5. Gifts (mismatch) | .18 | .20* | .23* | .26* | --- |  |
| 6. Relationship Satisfaction (women) | -.16 | -.30** | -.27** | -.23** | -.06 | --- |
| 7. Sexual Satisfaction (women) | .01 | -.35** | -.14 | -.23* | -.00 | .55** |
