# Supplementary material for: I love the way you love me: Responding to partner’s love language preferences boosts satisfaction in romantic heterosexual couples
Source: PLoS One. 2022 Jun 22;17(6):e0269429. doi: 10.1371/journal.pone.0269429 (PMC9216579; doi:10.1371/journal.pone.0269429)
Supplement: S1 File — (DOCX) [file pone.0269429.s003.docx]

Variables’ names and abbreviations

Dataset:

“*I love the way you love me: Responding to partner’s love language preferences boosts satisfaction in romantic heterosexual couples*”

**M** – Male;

**F** – Female;

**First_S_Intercourse** – the number of months that has pass since the first sexual intercourse with participant’s current partner;

**S_Amount_per_month** –an average number of sexual intercourses one has with a current partner per month;

**LL** – Love Language;

**LLS** – Love Languages Scale;

**RAS** – Relationship Assessment Scale;

**ISS** – Index of Sexual Satisfaction;

**IRI** – Interpersonal Reactivity Index;

**PT** – Perspective-taking scale;

**FS** – Fantasy scale;

**EC** – Empathic concern scale;

**PD** – Personal distress scale;

**Diff** – discrepancy scores indicating the degree of mismatch between the degree to which one prefers to feel love and one’s partner degree of expressing it in a following way;

**Abs** – converted discrepancy scores reflecting the degree of mismatch for each of the five LLs (i.e. the item-level sum of the four scores for each LL);

**Sum_Abs** – overall index of mismatch between one’s preferences for being loved and partner’s ways of expressing love, based on the 20 item-level discrepancy scores (i.e. summed discrepancy scores for each participant).
