# Supplementary material for: I love the way you love me: Responding to partner’s love language preferences boosts satisfaction in romantic heterosexual couples
Source: PLoS One. 2022 Jun 22;17(6):e0269429. doi: 10.1371/journal.pone.0269429 (PMC9216579; doi:10.1371/journal.pone.0269429)
Supplement: Attachment — Submitted filename: Response-to-Reviewers.docx [file pone.0269429.s004.docx]

**Subject:** Submission of revised paper entitled "I love the way you love me: Responding to partner’s love language preferences boosts satisfaction in romantic heterosexual couples”.

Dear Editors,

Thank you for your email dated April 5^th^, 2022 that addresses the points raised during the review process. We highly appreciate the Reviewer’s generous comments on the manuscript.

We have carefully reviewed them and revised the manuscript to address the following concerns, as well as the Journal Requirements.

We have addressed the editorial points as follows:

ad.1*: “Provide information about the dates on which the questionnaire was applied on the average time spent on responses (discuss whether there could have been condescending responses and other response biases), and whether there were several attention checks”.*

The questionnaire was applied over the period between October 2018 to March 2019. The subjects spent an average of 23 minutes responding to the questionnaire.

We have used the reverse wording items for each inventory to screen out careless respondents. No additional attention checks were used in the survey.

We are aware that self-response questionnaires tend to be affected by the response biases. We have clarified it in the Methods section and proposed an additional future research suggestion to address the potential flaws.

The information is now provided in the manuscript in lines 236-239, and 562-569.

ad. 2: *“Clarify if there were payments to participants as a way to encourage participation”.*

No, the subjects participated in the survey **voluntary** and were not paid for their participation. It is now clarified in the Method section and addressed as a potential limitation in the Discussion.

The information is now provided in the manuscript in lines 237-238, and 570-574.

ad. 3: *“In research in which self-response questionnaires are used, there could have been condescending responses and other response biases related to the social desirability…Please provide information about this topic on methods section”.*

We have provided information on the potential biases in response and participation in our survey, including the possibility of the condescending responses. It is now addressed in the Method and Discussion sections.

The information is now provided in the manuscript in lines 562-574, and addressed in 238-244.

ad. 4: *“Did you have any missing data or did everyone answer all the questions? If you did have missing data, how did you treat the "missingness"?”*

Yes. Ten couples were discarded from the study due to significant amount of missing information for one or both partners. When one question was missed (i.e. not scored) by the respondent, the average over available items was calculated and multiplied with the number of items in the questionnaire to replace the missing value.

The information is now provided in the manuscript in lines 215-222.

*Please change "female” or "male" to "woman” or "man" as appropriate, when used as a noun*

This point is now addressed across the entire manuscript; the only remaining uses of “male” and “female” are when these words are used as adjectives.

We have attached a marked-up copy that highlights changes made to the original version, as well as unmarked version of our revised manuscript. If you have any further questions or suggestions, please do not hesitate to contact us directly.

We hope that the revised version is now suitable for publication and look forward to hearing from you.

Thank you for your review of our paper.

Sincerely,
Dr. Maciej Stolarski.
